# Effect of exogenous xylanase, amylase, and protease as single or combined activities on nutrient digestibility and growth performance of broilers fed corn/soy diets

**DOI:** 10.3382/ps/pew297

**Published:** 2016-09-02

**Authors:** A. M. Amerah, L. F. Romero, A. Awati, V. Ravindran

**Affiliations:** * Danisco Animal Nutrition, DuPont Industrial Bioscience, Marlborough, SN8 1XN, UK; † Institute of Veterinary, Animal and Biomedical Sciences, Massey University, Palmerston North 4442, New Zealand

**Keywords:** broilers, exogenous enzymes, nutrient digestibility, performance

## Abstract

Two trials (a 42-d performance and a 21-d cohort digestibility) were conducted to evaluate the performance and nutrient digestibility of broilers fed corn diets supplemented with exogenous xylanase, amylase, and protease as single or combined activities. A nutritionally adequate, positive control (PC) diet was formulated. The negative control (NC) diet was formulated to be lower in metabolizable energy (∼86 kcal/kg diet) and digestible amino acids (1 to 2%) compared to PC. The other 4 treatments were based on the NC and they were either supplemented with xylanase (X), amylase (A), protease (P), or a combination of X, A, and P (XAP; to provide 2,000 U of X, 200 U of A, and 4,000 U of P/kg diet). All diets were marginal in AvP and Ca and contained a background of phytase (1,000 FTU/kg). In each trial, male broiler (Ross 308) chicks were allocated to the 5 treatments (10 replicates of 20 birds/pen and 9 replicates of 8 birds/cage for the performance and digestibility trials, respectively). In the digestibility trial, ileal digesta was collected on d21 for the determination of nutrient utilization. Data were subjected to one-way ANOVA and means were separated by Tukey's HSD test. Only the XAP improved (*P* < 0.05) AMEn compared to NC. X, A or XAP improved (*P* < 0.05) N digestibility and apparent ileal digestible energy (AIDE). Both P and XAP improved N retention. The relative improvement in energy digestibility due to enzyme supplementation was greater at the ileal level than that measured in the excreta. The measured changes on AIDE due to supplemental enzymes were much higher than the sum of calculated contributions from starch, fat, and protein. Supplementation of all enzymes reduced (*P* < 0.05) ileal flow of soluble rhamnose and mannose relative to NC. In the performance trial, both X and XAP improved (*P* < 0.05) weight gain (WG) and only XAP improved (*P* < 0.05) FCR compared to NC during the starter phase (1-21d). Over the entire period (1–42d), WG and FI were not influenced (*P* > 0.05) by dietary treatments. Both X and XAP had lower (*P* < 0.05) FCR compared to NC (1.540 and 1.509 vs 1.567, respectively). However, birds fed diet supplemented with XAP had an improved (*P* < 0.05) FCR compared to birds fed single activities and had similar (*P* > 0.05) FCR compared to PC. In conclusion, these results suggest a synergistic effect between X, A and P on broiler performance and nutrient digestibility. In the current study, AIDE measurements appeared to overestimate the enzyme response. Calculation of the energy contribution by supplemental enzymes using the improvements in the digestibility of the undigested fraction of starch, fat and protein may be a more accurate measurement for the enzyme response than the absolute response in AIDE.

## INTRODUCTION

Corn is the most commonly used grain in poultry diets worldwide and generally thought of as less variable compared to other grains such as wheat. However, Leeson et al. (1993) reported that metabolizable energy of 26 corn samples ranged from 2,926 to 3,473 kcal/kg from a relatively small geographic area. To manage variable corn quality and improve nutrient digestibility, exogenous enzymes such as xylanase (**X**), amylase (**A**), and protease (**P**) are increasingly being used in corn-based diets for broilers (Cowieson, 2010). There are several suggested modes of action for these enzymes (Adeola and Cowieson, 2011). For example, carbohydrases degrade cell wall components such as soluble and insoluble arabinoxylans, releasing encapsulated nutrients inside the cell wall and improve access of endogenous enzymes to cell contents (Cowieson, 2005). Enzymes such as A and P can augment endogenous digestive enzymes (Ritz et al., 1995; Gracia et al., 2003) and reduce endogenous amino acid (**AA**) losses through altering the production of pancreatic enzymes (Jiang et al., 2008) and mucin secretion (Cowieson and Bedford, 2009).

Multi-enzyme activity products have been used commercially in broiler diets for over 2 decades. Several studies have reported that a combination of X, A, and P improve broiler performance and nutrient digestibility in corn-based diets (Olukosi et al., 2007; Cowieson and Ravindran, 2008; Tang et al., 2014). Romero et al. (2013, 2014) recently reported the value of adding protease on top of X and A on energy metabolizability and nutrient digestibility. In contrast, Masey O’Neill et al. (2014) suggested that there is no evidence to support the use of multi-carbohydrase over single enzyme activity. There is scarcity of data showing the advantage of multi-component enzymes over a single-component activity. Therefore, the objective of this study was to examine the effect of exogenous X, A, and P as single or combined activities on nutrient digestibility and growth performance of broilers fed corn-based diets.

## MATERIALS AND METHODS

Experimental procedures were in accordance with the Massey University Animal Ethics Committee guidelines. The study involved 2 trials, namely a 42-d performance and a 21-d cohort digestibility.

### Exogenous Enzymes

The enzymes were endo-1,4-β-xylanase (EC 3.2.1.8), α-amylase (EC 3.2.1.1), and a serine protease (EC 3.4.21.62). The xylanase originated from *Trichoderma reesei*, the amylase from *Bacillus licheniformis* and the protease from *Bacillus subtilis*. The phytase used was a 6-phytase (EC 3.1.3.26) obtained from *Buttiauxella* sp. expressed in *Trichoderma reesei* (Axtra PHY, Danisco Animal Nutrition, DuPont Industrial Biosciences, Marlborough, UK). Enzymes activities in feed samples (200 g) were measured at the DuPont Nutrition Biosciences Innovation Laboratories (Brabrand, Denmark; Table 1) in duplicate. One xylanase unit was defined as the amount of enzyme that releases 0.48 μmol of reducing sugar as xylose from wheat arabinoxylan per minute at pH 4.2 and 50°C. One FTU was defined as the quantity of enzyme that releases 1 μmol of inorganic P/min from 5.0 mM sodium phytate at pH 5.5 at 37°C. One unit of *Bacillus licheniformis* α-amylase was the amount of enzyme required to release, in the presence of excess α-glucosidase, 0.20 μmol of glucosidic linkages expressed as p-nitrophenol equivalents from a maltoheptaoside substrate per minute at pH 8.0 and 40°C. One protease unit (PU) was defined as the amount of enzyme that releases 1.0 μg of phenolic compound, expressed as tyrosine equivalents, from a casein substrate per minute at pH 7.5 and 40°C.

**Table 1.** Expected and measured enzyme activities in feed samples.

	Xylanase^1^		Amylase^2^		Protease^3^		Phytase^4^	
	(XU/kg of feed)		(AU/kg of feed)		(PU/kg of feed)		(FTU/kg of feed)	

Dietary		Measured		Measured		Measured		Measured
treatment	Expected	(starter/finisher)	Expected	(starter/finisher)	Expected	(starter/finisher)	Expected	(starter/finisher)
PC	0	ND/ND	0	ND/ND	0	ND/ND	1,000	810/994
NC	0	ND/ND	0	ND/ND	0	ND/ND	1,000	1,037/1,223
NC + X	2,000	2,479/2,540	0	ND/ND	0	ND/ND	1,000	1,060/1,000
NC + A	0	ND/ND	200	218/236	0	ND/ND	1,000	1,258/1,148
NC + P	0	ND/ND	0	ND/ND	4,000	4,135/3,944	1,000	1,189/1,127
NC + XAP	2,000	3,076/2,415	200	182/332	4,000	4,581/4,072	1,000	1,189/1,229

X = xylanase from Trichoderma ressei (2,000 U/kg); A = amylase from Bacillus licheniformis (200 U/kg); P = protease from Bacillus subtilis (4,000 U/kg). ND = not detectable.

^1^XU: xylanase units defined as the amount of enzyme that releases 0.48 μmol of reducing sugar as xylose from wheat arabino xylan per minute at pH 4.2 and 50°C.

^2^AU: amylase units defined as the amount of enzyme required to release, in the presence of excess α-glucosidase, 0.20 μmol of glucosidic linkages expressed as *p*-nitrophenol equivalents from a maltoheptaoside substrate per minute at pH 8.0 and 40°C.

^3^PU: protease units defined as the amount of enzyme that releases 1.0 μg of phenolic compound, expressed as tyrosine equivalents, from a casein substrate per minute at pH 7.5 and 40°C.

^4^All diets contained 1,000 FTU of *Buttiauxella sp.* expressed in *Trichoderma reesei* phytase/kg of feed in the background. One FTU was defined as the quantity of enzyme that releases 1 μmol of inorganic P/min from 5.0 mM sodium phytate at pH 5.5 at 37°C.

### Diets and Treatments

Diets based on corn and soybean meal were formulated (Table 2) and fed over 2 phases (starter 1 to 21 d; grower/finisher 22 to 42 d) to meet Ross 308 strain nutrient recommendations for broiler (Ross, 2007). The negative control (**NC**) diet was formulated to be lower in metabolizable energy (∼86 kcal/kg diet) and digestible amino acids (1 to 2%) compared to the nutritionally adequate positive control (PC) diet. The other 4 treatments were based on the NC and were either supplemented with xylanase (X; 2,000 U/kg diet), amylase (A; 200 U/kg diet), protease (P; 4,000 U/kg diet) or a combination of X, A, and P (**XAP**; to provide 2,000 U of X, 200 U of A, and 4,000 U of P/kg diet; Axtra XAP, Danisco Animal Nutrition, DuPont Industrial Biosciences, Marlborough, UK). All diets contained phytase (1,000 FTU/kg), which was assumed to contribute 0.15% AvP and 0.14% Ca. For the digestibility trial, diets contained titanium dioxide (0.3%) as an inert marker. Diets for both trials were offered ad libitum and water was available at all times throughout the trial. All diets were pelleted at 70°C.

**Table 2.** Composition and calculated analysis (g/100 g as fed) of basal diets.^1^

	Starter (d 1–21)		Finisher (d 22–42)	

Ingredients	Positive control	Negative control	Positive control	Negative control
Corn	60.4	62.6	65.0	67.9
Soybean meal (48%)	32.9	32.2	27.8	26.7
Meat and bone meal	2.0	1.7	2.3	2.3
Poultry fat	1.84	0.30	2.7	0.81
L-lysine HCl	0.22	0.22	0.19	0.19
DL-methionine	0.34	0.33	0.27	0.26
L-threonine	0.08	0.07	0.05	0.04
Sodium bicarbonate	0.11	0.26	0.10	0.10
Salt	0.22	0.22	0.22	0.22
Limestone	1.04	1.12	0.93	0.94
Vitamin and trace mineral premix	0.30	0.30	0.30	0.30
Dicalcium phosphate	0.36	0.46	0.0	0.0
Phytase^2^	0.01	0.01	0.01	0.01
*Calculated analysis*				
Crude protein (%)	22.00	21.70	20.00	19.72
ME (kcal/kg)	3050	2966	3147	3059
Digestible lysine (%)	1.18	1.16	1.04	1.02
Digestible methionine (%)	0.62	0.61	0.53	0.52
Digestible M + C (%)	0.92	0.91	0.81	0.80
Calcium^3^ (%)	1.0	1.0	0.90	0.90
Available phosphorus^3^ (%)	0.45	0.45	0.40	0.40

^1^Supplied, per kilogram of diet: antioxidant, 100 mg; biotin, 0.2 mg; calcium pantothenate, 12.8 mg; cholecalciferol, 60 μg; cyanocobalamin, 0.017 mg; folic acid, 5.2 mg; menadione, 4 mg; niacin, 35 mg; pyridoxine, 10 mg; *trans*-retinol, 3.33 mg; riboflavin, 12 mg; thiamine, 3.0 mg; DL- *α*-tocopheryl acetate, 60 mg; choline chloride, 638 mg; Co, 0.3 mg; Cu,

3 mg; Fe, 25 mg; I, 1 mg; Mn, 125 mg; Mo, 0.5 mg; Se, 200 μg; Zn, 60 mg.

^2^Axtra PHY 10000 TPT, Danisco Animal Nutrition, Marlborough, UK. The enzyme was included at a rate of 100 g/t to supply a guaranteed minimum of 1,000 FTU/kg of feed.

^3^Includes the contribution from phytase of 0.14% Ca and 0.15% available P.

### Performance Trial

Day-old broilers (Ross 308) were obtained from a commercial hatchery, weighed, and housed in floor pens covered with wood shavings in an environmentally controlled room with 24 h fluorescent lighting. Each of the 5 dietary treatments was randomly assigned to 10 pens (20 birds/pen). Room temperature was maintained at 32 ± 1°C during the wk 1 and gradually decreased to 24°C by the end of wk 3. Body weight and feed intake (**FI**) were recorded on a pen basis at 21 and 42 d. Any bird that died was weighed and feed conversion ratio (**FCR**) values were calculated by dividing total feed intake by weight gain (**WG**) of live plus dead birds.

### Digestibility Trial

Day-old broilers (Ross 308) individually weighed and assigned to 45 cages (9 cages per treatment/8 birds per cage) in electrically heated battery brooders so that the average bird weight was similar for each cage. The birds were transferred to grower cages on d 12. The battery brooders and grower cages were housed in an environmentally controlled room with 20 h of fluorescent illumination daily. The temperature was maintained at 31°C on d 1 and gradually reduced to 22°C by 21 d of age. BW and FI were recorded by cage at 21 d of age. Mortality was recorded daily. Any bird that died was weighed and FCR values were calculated by dividing total feed intake by WG of live plus dead birds. On d 21, all birds were killed by intracardial injection of sodium pentobarbitone and contents of the lower ileum were expressed by gentle flushing with distilled water (Ravindran et al., 2005). The digesta samples were frozen immediately after collection, lyophilized and ground to pass through a 0.5-mm screen size and analyzed of dry matter, nitrogen, starch, fat, gross energy, non-starch polysaccharide (**NSP**) components, and titanium oxide.

Nitrogen-corrected apparent metabolizable energy (**AMEn**) was determined from d 17 to 20 posthatch; feed intake and total excreta output were measured quantitatively per cage over 4 consecutive days. Daily excreta collections were pooled within a cage, mixed in a blender, and subsampled. Each subsample was lyophilized, ground to pass through a 0.5-mm sieve, and stored in airtight plastic containers at 4°C pending analysis. The dry matter (**DM**) content, nitrogen, and gross energy were determined in replicate samples of diets and excreta.

### Chemical Analysis

The DM and crude fat were analyzed according to the procedures of Association of Official Analytical Chemists (AOAC, 2005). Nitrogen was determined using an FP-428 nitrogen determinator (LECO Corporation, St Joseph, MI). Gross energy (**GE**) was determined using an adiabatic oxygen calorimeter (Gallenkamp Autobomb, London, UK) standardized with benzoic acid. Starch was measured using an assay kit (Megazyme, Boronia, Victoria, Australia) based on the thermostable α-amylase and amyloglucosidase (McCleary et al., 1997). Analyses for the sugar components of NSP were done using the methods of Englyst et al. (1994). Titanium (**Ti**) was determined on a UV spectrophotometer following the method of Short et al. (1996).

### Calculations and Energy Contribution Calculations

The apparent ileal digestibility of DM, GE, N, starch, and fat were calculated by the following formula using the Ti marker ratios in the diet and ileal digesta (Ravindran et al., 2009).

\begin{eqnarray*}
\,\,\,\,{{\rm{Apparent}}\;{\rm{nutrient}}\;{\rm{digestibility}}\% =}\nonumber\\
\quad \left( {\left( {\left( {{\rm{NT}}/{\rm{Ti}}} \right){\rm{d}} - \left( {{\rm{NT}}/{\rm{Ti}}} \right){\rm{i}}} \right)/\left( {{\rm{NT}}/{\rm{Ti}}} \right){\rm{d}}} \right)*100
\end{eqnarray*}

where (NT/Ti)d = ratio of nutrient and Ti in diet and (NT/Ti)i = ratio of nutrient and Ti in ileal digesta.

Apparent ileal digestible energy (**AIDE**) was calculated by multiplying the diet GE content by the apparent ileal energy digestibility.

The apparent total tract retention coefficient (**ATTRC**) of nutrients was calculated using the following formula, with appropriate corrections for differences in dry matter content:

\begin{eqnarray*}
\,\,\,\,\,\,{{\rm{ATTRC}} = \big( {\rm{total}}\;{\rm{nutrient}}\;{\rm{ingested}}}\nonumber\\
\quad -\, {\rm{total}}\;{\rm{nutrient}}\;{\rm{excreted}} \big)/\left( {{\rm{total}}\;{\rm{nutrient}}\;{\rm{ingested}}} \right).\end{eqnarray*}

Nitrogen-corrected AME was calculated by correction for zero nitrogen retention by simple multiplication with 8.73 kcal/g of nitrogen retained in the body as described by Hill and Anderson (1958).


{\fontsize{6.7}{}\begin{eqnarray*}
% \fontsize{6.7}{8.7}\selectfont
\,\,\,\,\,\,{{\rm AME} \left( \rm{kcal}/\rm{kg\; diet} \right) = }\nonumber\\
{\frac{{(\rm{Feed\;intake \,{\times}\, GEdiet} ) {-} (\rm{Excreta\;output \,{\times}\, GEexcreta} )}}
{\rm{Feed\;intake}}}
\end{eqnarray*}
}

Calculations of the flow of NSP components were done using the concentrations of Ti in both the diet and the digesta as well as the concentration of the NSP component in digesta.

Apparent ileal digestible energy contributions from protein, starch, and fat in response to exogenous enzymes were calculated according to (Romero et al., 2014), using the following equation.

\[
{\rm{AIDEpsf}} = \left( {{\rm{AIDEU}} - {\rm{AIDNC}}} \right) \times {\rm{GEpsf}},
\]

where AIDEpsf was the apparent digestible energy contribution of each substrate (protein, starch, or fat; kcal/ kg of feed DM); AIDEU was the apparent ileal digestible substrate of each experimental unit (g/kg of feed DM); AIDNC was the arithmetic mean of the apparent ileal digestible substrate of the respective NC (g/kg of feed DM); and GEpsf was the GE density of each substrate (kcal/g). The GE density of protein was assumed to be 5.5 kcal/g; starch was assumed to contain 4.2 kcal/g; and fat was assumed to contain 9.1 kcal/g (Leeson and Summers, 2001).

### Data Analysis

Performance and nutrient digestibility data were analyzed by 1-way ANOVA using the GLM procedure of the JMP 11.0 software (SAS Institute Inc, 2013). Pen or cage means were considered as experimental units. A probability value of *P* < 0.05 was considered to be statistically significant. Means were separated by Tukey's HSD test. Mortality was analyzed using Pearson's Chi-square test to identify significant differences between treatments.

## RESULTS

### Performance Study

Pearson's Chi-square test showed no difference (*P* > 0.05) in the mortality between dietary treatments (Table 3). The effects of X, A and P as single or combined activities on WG, FI, FCR, calorie conversion and carcass characteristics in broilers are summarized in Table 3. During the starter phase (d 1 to 21), both X and XAP treatments improved (*P < *0.05); WG and XAP improved (*P* < 0.05) FCR compared to NC, and neither was significantly different from PC. No treatment effect (*P* > 0.05) was observed on broiler performance during the finisher phase (d 22 to 42). Over the entire period (d 1 to 42), WG and FI were not influenced (*P* > 0.05) by dietary treatments. Both X and XAP had lower (*P* < 0.05) FCR compared to NC (1.540 and 1.509 vs 1.567, respectively). However, birds fed the diet supplemented with XAP had lowest (*P* < 0.05) FCR compared to birds fed single activities and had similar (*P* > 0.05) FCR compared to PC. XAP had the most effective (*P* < 0.05) calorie conversion compared to other treatments. No treatment effect (*P* > 0.05) was observed for any of the measured carcass characteristics.

**Table 3.** Effect of exogenous xylanase, amylase, and protease as single or combined activities on weight gain (g/bird), feed intake (g/bird), feed conversion ratio (FCR, g/g), calorie conversion (kcal/kg body weight) and carcass characteristics (% of body weight) in broilers fed a corn-soy diet.^1^

	Positive control	Negative control (NC)	NC + X	NC + A	NC + P	NC + XAP	SEM^2^	*P*-value
**1-21 days**								
Weight Gain	1,114^a,b^	1,082^b^	1,131^a^	1,096^a,b^	1,102^a,b^	1,132^a^	36	0.001
Feed intake	1,347	1,353	1,387	1,357	1,365	1,370	36	0.17
FCR	1.209^c^	1.253^a^	1.227^a–c^	1.239^a–c^	1.241^a,b^	1.216^b,c^	0.01	0.0003
**22-42 days**								
Weight Gain	2,383	2,334	2,302	2,309	2,309	2,312	106	0.50
Feed intake	3,933	3,984	3,878	3,893	3,842	3,777	182	0.12
FCR	1.651	1.708	1.684	1.687	1.666	1.633	0.016	0.06
**1-42 days**								
Weight Gain	3,497	3,416	3,433	3,405	3,411	3,444	73	0.32
Feed intake	5,280	5,338	5,265	5,250	5,206	5,147	148	0.15
1-42 FCR	1.523^c,d^	1.567^a^	1.540^b,c^	1.548^a,b^	1.556^a,b^	1.509^d^	0.017	0.0001
Mortality (%)	7.0	6.0	5.5	6.5	8.0	6.5	1.6	0.22
Calorie conversion	4,731^a,b^	4,780^a^	4,690^a,b^	4,717^a,b^	4,672^b^	4,570^c^	48	0.002
**Carcass characteristics**								
Carcass weight	77.2	77.5	77.4	77.9	77.4	78.0	0.33	0.08
Leg	18.0	18.1	18.1	18.1	18.7	18.0	0.40	0.35
Abdominal fat	1.07	1.12	1.01	1.01	0.97	0.96	0.07	0.23
Breast meat	22.0	21.1	21.7	21.9	21.3	22.3	0.62	0.08

^a–d^Means in a row not sharing a common superscript are different (*P* < 0.05).

X = xylanase from *Trichoderma ressei* (2,000 U/kg); A = amylase from *Bacillus licheniformis* (200 U/kg); P = protease from *Bacillus subtilis* (4,000 U/kg).

^1^Each value represents the mean of 8 replicates (8 birds per replicate).

^2^Pooled standard error of the mean.

### Digestibility Study

The effects of X, A, and P as single or combined activities on AIDE, GE, N, starch, and fat digestibility are summarized in Table 4. X, A, and XAP improved (*P* < 0.05) the AIDE, GE and N digestibility compared to NC. All enzymes significantly improved starch digestibility compared to PC and NC. No treatment effect (*P* > 0.05) was observed for fat digestibility.

**Table 4.** Effect of exogenous xylanase, amylase, and protease as single or combined activities on apparent ileal digestible energy (AIDE) and ileal nutrient digestibility (%) in broilers fed a corn-soy diet (1 to 21 d posthatch).^1^

	AIDE	Starch	Nitrogen	Fat	GE
	kcal/kg DM	digestibility	digestibility	digestibility	digestibility
PC	3101^a–c^	96.7^b^	77.6^b,c^	89.9	69.8^b,c^
NC	2941^c^	96.9^b^	77.6^c^	87.3	67.9^c^
NC + X	3200^a,b^	97.7^a^	82.1^a^	91.3	73.9^a,b^
NC + A	3145^a,b^	98.0^a^	80.9^a,b^	89.9	72.6^a,b^
NC + P	3029^b,c^	97.6^a^	79.9^a–c^	90	69.9^b,c^
NC + XAP	3230^a^	98.1^a^	82.1^a^	90.3	74.6^a^
SEM^2^	69	0.25	1.1	1.07	1.6
*P* value	0.02	0.0002	0.01	0.11	0.01

^a–c^Means in a column not sharing a common superscript are different (*P* < 0.05).

X = xylanase from *Trichoderma ressei* (2,000 U/kg); A = amylase from *Bacillus licheniformis* (200 U/kg); P = protease from *Bacillus subtilis* (4,000 U/kg).

^1^Each value represents the mean of 9 replicates (8 birds per replicate).

^2^Pooled standard error of the mean.

The effects of X, A, and P as single or combined activities on AMEn, GE, N, and ash retention are summarized in Table 5. Single activities had no effect (*P* > 0.05) on the AMEn. The treatment with combined activities improved (*P* < 0.05) the AMEn compared to the NC. PC had the highest (*P* < 0.05) AMEn compared to other treatments. Energy retention was improved (*P* < 0.05) only by the XAP treatment. P and XAP treatments improved N retention compared to NC. Ash retention was not influenced (*P* > 0.05) by dietary treatments.

**Table 5.** Effect of exogenous xylanase, amylase, and protease as single or combined activities on nitrogen-corrected apparent metabolizable energy (AMEn, kcal/kg DM) and nutrient retention (g/kg DM intake) in broilers fed a corn-soy diet (1 to 21 d posthatch).^1^

	AMEn	Gross energy	Nitrogen	Ash
PC	3313^a^	79.3^b^	24.0^a^	29.1
NC	3217^c^	78.9^b^	22.8^c^	27.6
NC + X	3227^c^	79.1^b^	23.1^b,c^	32.9
NC + A	3231^b,c^	79.3^b^	23.3^b,c^	26.9
NC + P	3229^c^	79.2^b^	23.4^b^	28.0
NC + XAP	3267^b^	80.2^a^	23.4^b^	30.8
SEM^2^	10.5	0.25	0.19	2.4
P value	0.0001	0.01	0.003	0.50

^a–c^Means in a column not sharing a common superscript are different (*P* < 0.05).

X = xylanase from *Trichoderma ressei* (2,000 U/kg); A = amylase from *Bacillus licheniformis* (200 U/kg); P = protease from *Bacillus subtilis* (4,000 U/kg).

^1^Each value represents the mean of 9 replicates (8 birds per replicate).

^2^Pooled standard error of the mean.

The values of AIDE contribution of protein, starch, and fat due exogenous enzymes versus the NC treatment are shown in Table 6. The ileal flow of NSP components in response to the dietary treatments are shown in Tables 7 and 8. Supplementation of all enzymes reduced (*P* < 0.05) the ileal flow of soluble rhamnose and mannose relative to NC. Only X and XAP reduced (*P* < 0.05) ileal flow of total soluble NSP, galactose and galacturonic acid. Both X and XAP reduced (*P* < 0.05) ileal flow of insoluble fucose and galactose.

**Table 6.** Effect of exogenous xylanase, amylase, and protease as single or combined activities on the apparent ileal digestible energy (AIDE) contribution of protein, starch, and fat due exogenous enzymes versus the respective control treatments in broiler chickens at 21 d of age.^1^

	ADE contribution from	AIDE contribution from	AIDE contribution from	AIDEpsf^3^
	protein^2^ (kcal/kg of DM)	starch^2^ (kcal/kg of DM)	fat^2^ (kcal/kg of DM)	contribution (kcal/kg of DM)
NC + X^4^	40.3	14.5	7.5	62.3
NC + A^4^	23.7	16.0	5.7	45.4
NC + P^4^	27.5	13.2	9.3	50.0
NC + XAP	54.2	21.3	10.3	85.8

^1^The energy contribution of different substrates and AIDE were calculated as the difference of the observation in each experimental unit in the treatments fed enzymes versus the mean values of the negative control treatment.

^2^The calculated AIDE contribution from substrates was based on 1) the difference on the coefficient of ileal digestibility of the substrate versus that of the control treatments, 2) the nutrient content on the basal diets, and 3) a theoretical gross energy content of the substrates. The gross energy content of starch was assumed to be 4.2 kcal/g, fat was assumed to contain 9.1 kcal/g, and protein was assumed to contain 5.5 kcal/g.

^3^Sum of calculated AIDE contributions from protein, starch, and fat.

^4^X = xylanase from *Trichoderma ressei* (2,000 U/kg); A = amylase from *Bacillus licheniformis* (200 U/kg); P = protease from *Bacillus subtilis* (4,000 U/kg).

**Table 7.** Effect of exogenous xylanase, amylase, and protease as single or combined activities on ileal flow (g/kg dry matter intake) of components of soluble non-starch polysaccharides of broilers fed a corn-soy based diet (1 to 21 d posthatch).^1^

Treatment	rha	fuc	ara	xyl	man	gal	glu	GlcA	GalA	Total
PC	0.51^a,b^	1.07	4.47	1.40	1.60^a,b^	10.70^a^	1.91	0.41	5.78^a,b^	27.02^a,b^
NC	0.61^a^	1.17	5.29	1.43	1.81^a^	11.22^a^	2.35	0.38	6.19^a^	30.16^a^
NC + X	0.39^b,c^	0.84	3.94	1.35	1.23^c^	8.01^c^	1.44	0.42	4.72^c^	22.35^b^
NC + A	0.35^b,c^	1.02	4.88	1.65	1.47^b,c^	10.09^a,b^	1.67	0.41	5.19^a–c^	26.7^a,b^
NC + P	0.25^c^	1.20	4.62	1.26	1.46^b,c^	9.82^a,b^	1.82	0.46	5.77^a,b^	26.57^a,b^
NC + XAP	0.41^b,c^	0.87	4.56	1.79	1.29^c^	8.69^b,c^	1.71	0.34	4.63^c^	23.19^b^
SEM^2^	0.07	0.08	0.38	0.29	0.09	0.63	0.30	0.12	0.37	1.7
*P* value	**0.01**	0.06	0.27	0.78	**0.001**	**0.01**	0.49	0.99	**0.04**	**0.03**

^a–c^Means in a column not sharing a common superscript are different (*P* < 0.05).

X = xylanase from *Trichoderma ressei* (2,000 U/kg); A = amylase from *Bacillus licheniformis* (200 U/kg); P = protease from *Bacillus subtilis* (4,000 U/kg). rha, rhamnose; fuc, fucose; ara, arabinose; xyl, xylose; man, mannose; gal, galactose, glu, glucose; GlcA, glucuronic acid; GalA, galacturonic acid.

^1^Each value represents the mean of 9 replicates (8 birds per replicate).

^2^Pooled standard error of the mean.

**Table 8.** Effect of exogenous xylanase, amylase, and protease as single or combined activities on ileal flow (g/kg dry matter intake) of components of insoluble non-starch polysaccharides of broilers fed a corn-soy based diet (1 to 21 d posthatch).^1^

Treatment	rha	fuc	ara	xyl	man	gal	glu	GlcA	GalA	Total
PC	0.57	0.78^a^	16.03	17.00	0.89	13.45^a^	22.73	0.59	4.12	109
NC	0.54	0.76^a^	16.55	17.99	0.79	13.47^a^	23.52	0.59	3.65	112
NC + X	0.41	0.58^c^	15.14	17.31	0.81	10.64^b^	21.19	0.51	3.09	105
NC + A	0.56	0.68^a–c^	14.80	18.00	0.88	11.67^a,b^	21.73	0.53	3.07	103
NC + P	0.55	0.73^a,b^	15.80	17.41	0.83	12.63^a,b^	22.40	0.54	3.48	108
NC + XAP	0.45	0.61^b,c^	14.23	15.17	0.71	11.31^b^	20.50	0.53	3.02	93
SEM^2^	0.04	0.04	0.85	0.85	0.06	0.69	1.21	0.15	0.58	6.1
*P* value	0.13	**0.01**	0.22	0.51	0.51	**0.03**	0.51	0.99	0.75	0.29

^a–c^Means in a column not sharing a common superscript are different (*P* < 0.05).

X = xylanase from *Trichoderma ressei* (2,000 U/kg); A = amylase from *Bacillus licheniformis* (200 U/kg); P = protease from *Bacillus subtilis* (4,000 U/kg). rha, rhamnose; fuc, fucose; ara, arabinose; xyl, xylose; man, mannose; gal, galactose, glu, glucose; GlcA, glucuronic acid; GalA, galacturonic acid.

^1^Each value represents the mean of 9 replicates (8 birds per replicate).

^2^Pooled standard error of the mean.

## DISCUSSION

The objective of this study was to examine the effect of exogenous X, A, and P as single or combined activities on nutrient digestibility and growth performance of broilers fed corn-based diets. Xylanase supplementation improved the FCR compared to the NC during the starter phase and over the entire period (1-42d). The main substrate for the X in corn-soy diets is the insoluble arabinoxylan. Corn contains 1 g/kg soluble arabinoxylan and 51 g/kg insoluble arabinoxylan which is the main endosperm cell wall component (Choct, 2006; Taylor et al., 2013). Xylanase may increase the access of endogenous and exogenous enzymes to protein and starch within the endosperm cell (Cowieson, 2005) by breaking down the highly branched insoluble arabinoxylans in the cell wall (Chesson, 2001). It may also produce fermentable xylo-oligosaccharides (Fernandez et al., 2000) which are fermented to volatile fatty acids in the ceca. This will have positive effects on gut health and enhance digestion and absorption in the small intestine through peptide YY production (Masey O’Neill et al., 2012; Singh et al., 2012) which results in delayed gastric emptying and duodenal transit rates (Cuche et al., 2000; Park et al., 2013). It has been suggested that X may reduce mucin secretion as a result of breaking down fiber that irritates the gut lining (Cowieson and Bedford, 2009). This hypothesis in chickens is not conclusive (Sharma et al., 1997; Fernandez et al., 2000). Recently, Kiarie et al. (2014) reported that X improved growth performance and AMEn in both wheat- and corn-based diets suggesting the hydrolysis of both soluble and insoluble NSP. Similar results in corn-soy diets have been reported by others (Cowieson, 2010; Masey O’Neill et al., 2011; 2012) which support the use of X in corn-based diets.

On the other hand, the addition of single activities of amylase or protease resulted in intermediate effect on broiler performance. The FCR of these 2 treatments was not significantly different from single X or the NC. Gracia et al. (2003) reported that exogenous A supplementation to corn-soy diets improved broiler performance at all ages (7, 21, and 42 d) and increased the digestibility of starch and the AMEn. Similarly, Jiang et al. (2008) reported that A significantly improved WG by 4.5% and FI by 3.6%. In contrast, Kaczmarek et al. (2014) reported no effect of A on growth performance of chickens fed a conventional corn-containing diet (corn geometric mean diameters of 736 μm), but improved WG, FCR, and diet AMEn in those fed the finely ground corn (corn geometric mean diameters of 482 μm), possibly due to increased starch digestion in the upper gut. It was suggested that exogenous A augmented pancreatic A activity (Gracia et al., 2003). Mahagna et al. (1995) reported that secretion of endogenous A and P by the pancreas was reduced when birds were fed diets supplemented with A and P. Jiang et al. (2008) found that the mean pancreatic level of A activity was reduced from 9% to 33% with increasing levels of exogenous A from 250 mg/kg to 2,250 mg/kg as a result of feedback mechanism. Therefore, one of the modes of action for exogenous A and P is probably through decreasing endogenous AA losses which constitute an important part of the gut maintenance costs. This may explain, partly, the slightly better FCR in these 2 treatments. The effect of amylase on broiler performance may depend on enzyme bio-efficacy, corn quality (flint or dent), particle size, growing condition, drying conditions, level of resistance starch, and the ratio of amylose to amylopectin.

Protease has been used in broiler diets for almost 20 years (Bedford et al., 1997). Recently, there has been a renewed interest in the concept of protease supplementation of broiler diets, which is driven mainly by the increasing cost of ingredient and environmental concerns. The effect of protease on broiler performance and nutrient digestibility, however, has been inconsistent (Naveed et al., 1998; Ghazi et al., 2003; Rutherfurd et al., 2007; Boguhn and Rodehutscord, 2010; Angel et al. 2011; Romero et al., 2013, 2014). Angel et al. (2011) reported improvements in WG, FCR, and digestibility of specific AA at an inclusion of 200 mg/kg (15,000 PROT/kg), which was greater compared to a NC. In agreement, Olukosi et al. (2015) reported that single protease increased the AID of N particularly at the high enzyme dose (10,000 PU/kg). When adding the P on top of a X and A combination, they reported a further improvement in N digestibility by 2.1% points compared to P alone. The results of the current study showed that P alone numerically improved N digestibility by 2.3% points and the combination of X, A, and P elicited an additional numerical improvement of 2.2% points compared to P alone treatment. It should be noted, however, that the response to exogenous enzymes on AA digestibility was reported to be dependent on the inherent digestibility of the diet (Romero et al., 2013) and may also be related to the physical or chemical structure of the cereal grains (Amerah, 2015). Interestingly, X and A as single activity or the combination of X, A, and P significantly improved N digestibility compared to the NC. Romero et al. (2013) reported that P on top of X and A combination further increased AID of AA and AMEn in young broilers. Romero et al. (2014) also reported that the X and A combination and the X, A, and P combination treatments gradually increased AID of N at 21 d, but only X, A, and P combination increased AID of N compared with the control at 42 d. In the current study, the combination of X, A, and P improved GE retention compared to other treatments and it was the only treatment that improved AMEn compared to the NC. X, A, and the combination of X, A, and P improved AIDE. In contrast, Cowieson and Adeola (2005) reported no improvement in AIDE after supplementation of a corn/soy diet with a combination of X, A, and P, and phytase, but reported a significant increase in AIDE if the phytase was not included. It should be noted, however, that phytase (1,000 FTU/kg) was included in the background of all diets in the current study. The response to enzymes on energy improvement may vary depending on the energy content of the basal diet, the substrate level and inherent digestibility, gut health and the effect of enzymes on the microbiota profile. All enzyme treatments improved starch digestibility compared to control treatments, but fat digestibility was not influenced by dietary treatments. These results are in agreement with Romero et al. (2014) who reported that XA and XAP combinations improved starch digestibility but had no effect on fat digestibility in corn-/soy-based diets.

In the current study, the XAP treatment improved the FCR similar to that of the PC and had the best calorie conversion ratio compared to other treatments. These results are suggestive of synergism between X, A, and P. To our knowledge, this is the first study that compared the effect of single activities of X, A, and P and the combination of these enzymes. Although each enzyme targets a specific substrate, the synergistic effects of using these enzymes in combination may be due to the fact that the effect of the enzyme exceeds the response to their specific substrate. For example, by disrupting the cell wall with the X, protein and starch digestibility can be improved by making the nutrients within the cell more accessible to other enzymes. Protease may support the effect of X by breaking down the cell wall structure and play a role in the solubilization and disappearance of fiber (Zyla et al., 1999; Olukosi et al., 2015). Both A and P may disrupt the starch protein interaction within the cell and improve the digestion of both nutrients (Amerah, 2015). Dietary X, alone and in combination with other exogenous enzymes such as P and A, have reportedly improved growth performance, enhanced flock uniformity, improved energy and nutrient availability, and reduced nutrient excretion in broiler chickens (Zanella et al., 1999; Cowieson and Ravindran, 2008; Adeola and Cowieson, 2011).

When comparing the AMEn of the diets to AIDE, the AMEn values were higher. The difference between these 2 values is likely to be related to microbial fermentation in the hindgut. Interestingly X, A, and the combination of X, A, and P reduced the difference between AMEn and the AIDE to 27, 86, and 37 kcal/kg DM, respectively, compared to a difference of 212, 276, and 200 kcal/kg DM, for PC, NC, and P treatments, respectively. These data suggest less substrates being available for fermentation in the lower part of the digestive tract when carbohydrases are included. The relative improvement in energy digestibility compared to the NC due to enzyme supplementation at the ileal level was greater than that measured in the excreta. For example, the combination of X, A, and P supplementation improved the AIDE by 289 kcal/kg (9.8%) but AMEn was improved by only 50 kcal/kg (1.6%). Similar results were observed by Cowieson and Ravindran (2008). These researchers attributed these findings to the modifying effects of enzymes on hindgut microbiota and suggested that the microbial fermentation may lead to underestimation of the true effect of the enzyme product and therefore the ileal measurements may be a more accurate method to detect enzyme effects than on a total tract basis. The largest contributor to the AIDE improvement with enzyme supplementation was the protein fraction with only a marginal contribution of fat digestibility (Table 6). Romero et al. (2014) reported similar results with corn-based diets in broilers at 21d of age. The measured changes on AIDE due to supplemental enzymes were much higher than the sum of calculated contributions from starch, fat, and protein. In contrast, Romero et al. (2014) found only minor differences between the measured and calculated energy values in a corn-soy diet which was attributed the sum of experimental errors from the measurement of substrates in diet, digesta, and the inert marker. The observed large differences between the measured and calculated energy are difficult to explain. The obvious substrates contributing to this difference are NSP. The ileal flow of total soluble NSP of X and the XAP combination treatments was lower compared to the NC. Both X and the X, A, and P combination significantly reduced soluble and insoluble galactose, and insoluble fucose. All enzyme treatments reduced the flow of soluble rhamnose and mannose, whereas xylose was not influenced. These results confirm the complexity and the interaction between the different components within the diet. A close relationship between fiber and protein has been reported in corn (Rybka et al., 1992; Parker et al., 1999). In contrast to the current study, Olukosi et al. (2015) reported that protease alone reduced the flow of insoluble arabinose but did not affect the flow of insoluble xylose. They also reported that the combination of XAP reduced the ileal flow of insoluble and total glucose and galactose. Romero et al. (2014) suggested that enzyme supplementation may have caused either an increased fermentation of NSP or the absorption of pentose sugars in the small intestine which may explain the differences between measured and calculated energy. It appears that fiber plays a role in energy measurement at the ileal level and the response in AIDE cannot be attributed only to improvements in the digestion and absorption of starch, fat and protein. Recently, de Vries et al. (2014) suggested that the marker method is unsuitable to measure ileal fiber digestibility in broilers due to fractionation of soluble and solid digesta fractions. Such fractionation of soluble and solid digesta fractions, may partly explain the unexplainable high improvement in IDE due to enzyme supplementation. It is possible that enzymes influenced the NSP solubility and reduced transit time in the digestive tract relevant to the indigestible marker which resulted in overestimation of the enzyme energy improvement response. It is noteworthy that the expected enzyme response (energy difference between the PC and NC) which was calculated based on the expected improvement in the digestibility of ileal undigested fraction of protein, starch and fat is similar to actual measured in this study (84 kcal/kg). Therefore, it appears that energy calculation based on the improvement in the digestibility of the ileal undigested fraction of protein, starch, and fat due to exogenous enzymes may be a more accurate method to detect enzyme effects than absolute response in AIDE.

The number of studies examining the effect of enzyme combination on carcass characteristics is limited. No treatment effect on carcass characteristics was observed in this study. Similarly, Zanella et al. (1999) reported no effect of X, A, and P combination on carcass weight, abdominal fat and breast meat weight. Café et al. (2002) reported that abdominal fat was consistently increased by the X, A, and P combination and suggested that birds fed the diets containing enzymes obtained a greater amount of net energy from their diets.

In conclusion, our results showed positive effects of the enzyme combination on broiler performance compared to single enzyme activities, suggesting synergism between X, A, and P. In the current study, AIDE measurements appeared to overestimate the enzyme response. Calculation of the energy contribution by supplementing enzymes using the improvements in the digestibility of the undigested fraction of starch, fat, and protein may be a more accurate measurement for the enzyme response than absolute response in AIDE.

## References

[bib1] Adeola O. , Cowieson A. J.. 2011. Opportunities and challenges in using exogenous enzymes to improve non-ruminant animal production. J. Anim. Sci. 89:3189–3218. 21512114 10.2527/jas.2010-3715

[bib2] Amerah A. M. 2015. Interactions between wheat characteristics and feed enzyme supplementation in broiler diets. Anim. Feed Sci. Technol. 199:1–9.

[bib3] Angel C. R. , Saylor W., Vieira W. L., Ward N.. 2011. Effects of a monocomponent protease on performance and protein utilization in 7- to 22-day-old broiler chickens. Poult. Sci. 90:2281–2286. 21934011 10.3382/ps.2011-01482

[bib4] Association of Official Analytical Chemists (AOAC). 2005. Official methods of analysis, 15th edition. AOAC, Arlington, VA.

[bib5] Bedford M. R. , Morgan A. J., Clarkson K., Schultze H. K., 1997. Enzyme feed additive and animal feed. US Patent 5612055.

[bib6] Boguhn J. , Rodehutscord M.. 2010. Effects of nonstarch polysaccharide-hydrolyzing enzymes on performance and amino acid digestibility in turkeys. Poult. Sci. 89:505–513. 20181867 10.3382/ps.2009-00321

[bib6a] Café M. B. , Borges C. A., Fritts C. A., Waldroup P. W. Avizyme improves performance of broilers fed corn-soybean meal-based diets J. Appl. Poult. Res. 2002 11 29–33.

[bib7] Chesson A. 2001. Non-starch polysaccharide degrading enzymes in poultry diets: Influence of ingredients on the selection of activities. World Poult. Sci. J. 57:251–263.

[bib8] Choct M. 2006. Enzymes for the feed industry: past, present and future. World's Poult. Sci. J. 62:5–16.

[bib9] Cowieson A. J. 2005. Factors that affect the nutritional value of maize for broilers. Anim. Feed Sci. Technol. 119:293–305.

[bib10] Cowieson A. J. 2010. Strategic selection of exogenous enzymes for corn/soy-based poultry diets. Jpn. Poult. Sci. 47:1–7.

[bib11] Cowieson A. J. , Adeola O.. 2005. Carbohydrases, protease, and phytase have an additive beneficial effect in nutritionally marginal diets for broiler chicks. Poult. Sci. 84:1860–1867. 16479942 10.1093/ps/84.12.1860

[bib12] Cowieson A. J. , Bedford M. R.. 2009. The effect of phytase and carbohydrase on ileal amino acid digestibility in monogastric diets: Complimentary mode of action? World Poult. Sci. J. 65:609–624.

[bib13] Cowieson A. J. , Ravindran V.. 2008. Effect of exogenous enzymes in maize-based diets varying in nutrient density for young broilers: Growth performance and digestibility of energy, minerals and amino acids. Br. Poult. Sci. 49:37–44. 18210288 10.1080/00071660701812989

[bib14] Cuche G. , Cuber J. C., Malbert H.. 2000. Ileal short-chain fatty acids inhibit gastric motility by a humoral pathway. Am. J. Physiol. Gastro. Liver Physiol. 279:G925–G930. 10.1152/ajpgi.2000.279.5.G925 11052989

[bib15] de Vries S. , Kwakkel R. P., Pustjens A. M., Kabel M. A., Hendriks W. H., Gerrits W. J.. 2014. Separation of digesta fractions complicates estimation of ileal digestibility using marker methods with Cr2O3 and cobalt-ethylenediamine tetraacetic acid in broiler chickens. Poult. Sci. 93:2010–2017. 24931962 10.3382/ps.2013-03845

[bib16] Englyst H. N. , Quigley M. E., Hudson G. J.. 1994. Determination of dietary fiber as non-starch polysaccharides with gas-liquid chromatographic, high-performance liquid chromatographic or spectrophotometric measurement of constituent sugars. Analyst. 119:1497–1509. 7943740 10.1039/an9941901497

[bib17] Fernandez F. , Sharma R., Hilton M., Bedford M. R.. 2000. Diet influences the colonization of Campylobacter jejuni and distribution of mucin carbohydrates in the chick intestinal tract. Cell. Mol. Life Sci. 57:1793–1801. 11130183 10.1007/PL00000659 PMC11146916

[bib18] Ghazi S. , Rooke J. A., Galbraith H.. 2003. Improvement of the nutritive value of soybean meal by protease and α-galactosidase treatment in broiler cockerels and broiler chicks. Br. Poult. Sci. 44:410–418. 12964625 10.1080/00071660310001598283

[bib19] Gracia M. I. , Araníbar M. J., Lázaro R., Medel P., Mateos G. G.. 2003. Alpha-amylase supplementation of broiler diets based on corn. Poult. Sci. 82:436–442. 12705405 10.1093/ps/82.3.436

[bib20] Hill F. W. , Anderson D. L.. 1958. Comparison of metabolizable energy and productive energy determinations with growing chicks. J. Nutr. 64:587–603. 13549992 10.1093/jn/64.4.587

[bib21] Jiang Z. , Zhou Y., Lu F., Han Z., Wang T.. 2008. Effects of different levels of supplementary alpha-amylase on digestive enzyme activities and pancreatic amylase mRNA expression of young broilers. Asian-Austral. J. Anim. Sci. 21:97–102.

[bib22] Kaczmarek S. A. , Rogiewicz A., Mogielnicka M., Rutkowski A., Jones R. O., Slominski B. A.. 2014. The effect of protease, amylase, and non-starch polysaccharide-degrading enzyme supplementation on nutrient utilization and growth performance of broiler chickens fed corn-soybean meal-based diets. Poult. Sci. 93:1745–1753. 24864284 10.3382/ps.2013-03739

[bib23] Kiarie E. , Romero L. F., Ravindran V.. 2014. Growth performance, nutrient utilization, and digesta characteristics in broiler chickens fed corn or wheat diets without or with supplemental xylanase. Poult. Sci. 93:1186–1196. 24795311 10.3382/ps.2013-03715

[bib24] Leeson S. , Summers J. D.. 2001. Energy. Pages 34–99 in Nutrition of the Chicken. 4th ed. University Books. Guelph, ON, Canada.

[bib25] Leeson S. , Yersin A., Volker L.. 1993. Nutritive value of the 1992 maize crop. J. Appl. Poult. Res. 2:208–213.

[bib26] Mahagna M. , Nir I., Larbier M., Nitsan Y.. 1995. Effect of age and exogenous amylase and protease on development of the digestive tract, pancreatic enzyme activities and digestibility of nutrients in young meat-type chicks. Reprod. Nutr. Dev. 35:201–212 7537505 10.1051/rnd:19950208

[bib27] Masey O’Neill H. V. , Smith J. A., Bedford M. R.. 2014. Multicarbohydrase enzymes for non-ruminants. Asian-Austral. J. Anim. Sci. 27:290–301. 10.5713/ajas.2013.13261 PMC4093217 25049954

[bib28] Masey O’Neill H. V. , Lui N., Wang J. P., Diallo A., Hill S.. 2011. Effect of xylanase on performance and apparent metabolisable energy in starter broilers fed diets containing one maize variety harvested in different regions of china. Asian-Austral. J. Anim. Sci. 25:515–523. 10.5713/ajas.2011.11314 PMC4092908 25049592

[bib29] Masey O’Neill H. V. M. , Haldar S., Bedford M. R.. 2012. The role of peptide YY in the mode of action of dietary xylanase. Poult. Sci. 91:217.

[bib30] McCleary B. V. , Gibson T. S., Mugford D. C.. 1997. Measurement of total starch in cereal products by amyloglucosidase vs. amylase method: collaborative study. J. Assoc. Off. Anal. Chem. 80:571–579.

[bib31] Naveed A. , Acamovic T., Bedford M. R.. 1998. Effect of enzyme supplementation of UK-known Lupinus albus on growth performance in broiler chickens. Br. Poult. Sci. 39:S36–S37. 10188036 10.1080/00071669888250

[bib32] Olukosi O. A. , Beeson L. A., Englyst K., Romero L. F.. 2015. Effects of exogenous proteases without or with carbohydrases on nutrient digestibility and disappearance of non-starch polysaccharides in broiler chickens. Poult. Sci. 94:2662–2669. 26371327 10.3382/ps/pev260 PMC4988624

[bib33] Olukosi O. A. , Cowieson A., Adeola O.. 2007. Age-related influence of a cocktail of xylanase, amylase, and protease or phytase individually or in combination in broilers. Poult. Sci. 86:77–86. 17179419 10.1093/ps/86.1.77

[bib34] Park J. H. , Won O. K., Ahn S. H., Lee S., Choi B. K., Jung K. Y.. 2013. Fatty diets retarded the propulsive function of and attenuated motility in the gastrointestinal tract of rats. Nutr. Res. 33:228–234. 23507229 10.1016/j.nutres.2012.12.008

[bib35] Parker M. L. , Grant A., Rigby N. M., Belton P. S., Taylor J. R. N.. 1999. Effects of popping on the endosperm cell walls of sorghum and maize. J. Cereal Sci. 30:209–216.

[bib36] Ravindran V. , Hew L. I., Ravindran G., Bryden W. L.. 2005. Apparent ileal digestibility of amino acids in dietary ingredients for broiler chickens. Anim. Sci. 81:85–97.

[bib37] Ravindran V , Morel P. C. H., Rutherfurd S. M., Thomas D. V.. 2009. Endogenous flow of amino acids in the avian ileum is increased by increasing dietary peptide concentrations. Br. J. Nut. 101:822–828. 10.1017/S0007114508039974 18662428

[bib38] Ritz C. W. , Hulet R. M., Self B. B., Denbow D. M.. 1995. Growth and intestinal morphology of male turkeys as influenced by dietary supplementation of amylase and xylanase. Poult. Sci. 74:1329–1334. 7479512 10.3382/ps.0741329

[bib39] Romero L. F. , Parsons C. M., Utterback P. L., Plumstead P. W., Ravindran V.. 2013. Comparative effects of dietary carbohydrases without or with protease on the ileal digestibility of energy and amino acids and AMEn in young broilers. Anim. Feed Sci. Technol. 181:35–44.

[bib40] Romero L. F. , Sands J. S., Indrakumar S. E., Plumstead P. W., Dalsgaard S., Ravindran V.. 2014. Contribution of protein, starch, and fat to the apparent ileal digestible energy of corn- and wheat-based broiler diets in response to exogenous xylanase and amylase without or with protease. Poult. Sci. 93:1–13. 25071229 10.3382/ps.2013-03789 PMC4988540

[bib41] Ross. 2007. Ross 308 Broiler: Nutrition Specifications, June 2007. Ross Breeders Limited, Newbridge, Midlothian, Scotland, UK.

[bib42] Rutherfurd S. M. , Chung T. K., Moughan P. J.. 2007. The effect of a commercial enzyme preparation on apparent metabolizable energy, the true ileal amino acid digestibility, and endogenous ileal lysine losses in broiler chickens. Poult. Sci. 86:665–672. 17369537 10.1093/ps/86.4.665

[bib43] Rybka K. , Boros D., Raczynska-Bojanowska K.. 1992. Comparative studies on the components of cereal grain that are undigestible in vitro. J. Cereal Sci. 15:295–303.

[bib44] Singh A. , O’Neill H. V. M., Ghosh T. K., Bedford M. R., Haldar S.. 2012. Effects of xylanase supplementation on performance, total volatile fatty acids and selected bacterial population in caeca, metabolic indices and peptide YY concentrations in serum of broiler chickens fed energy restricted maize–soybean based diets. Anim. Feed Sci. Technol. 177:194–203.

[bib7a] SAS Institute Inc. Using JMP 11, 2013 Cary, NC: SAS Institute Inc..

[bib45] Sharma R. , Fernandez F., Hilton M., Schumacher U.. 1997. The influence of diet on the mucin carbohydrates in the chicken tract. Cell. Mol. Life Sci. 53:935–942. 9447246 10.1007/s000180050114 PMC11147344

[bib46] Short F. J. , Gorton P., Wiseman J., Boorman K. N.. 1996. Determination of titanium dioxide added as an inert marker in chicken digestibility studies. Anim. Feed Sci. Technol. 59:215–221.

[bib47] Tang D. , Hao S., Liu G., Nian F., Ru Y.. 2014. Effects of maize source and complex enzymes on performance and nutrient utilization of broilers. Asian-Austral. J. Anim. Sci. 27:1755–1762. 10.5713/ajas.2014.14255 PMC4213688 25358370

[bib48] Taylor J. R. N. , Dlamini B. C., Kruger J., 2013. The science of the tropical cereals sorghum, maize and rice in relation to lager beer brewing. J. Inst. Brew. 119, 1e14.

[bib49] Zanella I. , Sakomura N. K., Silversides F. G., Figueiredo A., Pack M.. 1999. Effect of enzyme supplementation of broiler diets based on corn and soybeans. Poult. Sci. 78:561–568. 10230910 10.1093/ps/78.4.561

[bib50] Zyla K. , Gogol D., Koreleski J., Swiatkiewicz S., Ledoux D. R.. 1999. Simultaneous application of phytase and xylanase to broiler feeds based on wheat: in vitro measurements of phosphorus and pentose release from wheats and wheat-based feeds. J. Sci. Food Agric. 79:1832–1840.

